# CDK14 Promotes Axon Regeneration by Regulating the Noncanonical Wnt Signaling Pathway in a Kinase-Independent Manner

**DOI:** 10.1523/JNEUROSCI.0711-21.2021

**Published:** 2021-10-06

**Authors:** Naoki Hisamoto, Yoshiki Sakai, Kohei Ohta, Tatsuhiro Shimizu, Chun Li, Hiroshi Hanafusa, Kunihiro Matsumoto

**Affiliations:** Division of Biological Science, Graduate School of Science, Nagoya University, Nagoya 464-8602, Japan

**Keywords:** axon regeneration, *C. elegans*, cdk14, noncanonical Wnt signaling pathway

## Abstract

The postinjury regenerative capacity of neurons is known to be mediated by a complex interaction of intrinsic regenerative pathways and external cues. In *Caenorhabditis elegans*, the initiation of axon regeneration is regulated by the nonmuscle myosin light chain-4 (MLC-4) phosphorylation signaling pathway. In this study, we have identified *svh-16*/*cdk-14*, a mammalian CDK14 homolog, as a positive regulator of axon regeneration in motor neurons. We then isolated the CDK-14-binding protein MIG-5/Disheveled (Dsh) and found that EGL-20/Wnt and the MIG-1/Frizzled receptor (Fz) are required for efficient axon regeneration. Further, we demonstrate that CDK-14 activates EPHX-1, the *C. elegans* homolog of the mammalian ephexin Rho-type GTPase guanine nucleotide exchange factor (GEF), in a kinase-independent manner. EPHX-1 functions as a GEF for the CDC-42 GTPase, inhibiting myosin phosphatase, which maintains MLC-4 phosphorylation. These results suggest that CDK14 activates the RhoGEF–CDC42–MLC phosphorylation axis in a noncanonical Wnt signaling pathway that promotes axon regeneration.

**SIGNIFICANCE STATEMENT** Noncanonical Wnt signaling is mediated by Frizzled receptor (Fz), Disheveled (Dsh), Rho-type GTPase, and nonmuscle myosin light chain (MLC) phosphorylation. This study identified *svh-16*/*cdk-14*, which encodes a mammalian CDK14 homolog, as a regulator of axon regeneration in *Caenorhabditis elegans* motor neurons. We show that CDK-14 binds to MIG-5/Dsh, and that EGL-20/Wnt, MIG-1/Fz, and EPHX-1/RhoGEF are required for axon regeneration. The phosphorylation-mimetic MLC-4 suppressed axon regeneration defects in *mig-1*, *cdk-14*, and *ephx-1* mutants. CDK-14 mediates kinase-independent activation of EPHX-1, which functions as a guanine nucleotide exchange factor for CDC-42 GTPase. Activated CDC-42 inactivates myosin phosphatase and thereby maintains MLC phosphorylation. Thus, the noncanonical Wnt signaling pathway controls axon regeneration via the CDK-14–EPHX-1–CDC-42–MLC phosphorylation axis.

## Introduction

The ability of axons to regenerate after an injury is a fundamental and conserved property of neurons, governed by interactions between the intrinsic growth state and the extracellular microenvironment (Kaplan et al., 2015). Neurons in the mammalian peripheral nervous system, like neurons in most invertebrates, retain the ability to regenerate. In contrast, neurons in the mammalian CNS have a limited regenerative capacity (Case and Tessier-Lavigne, 2005). This difference in regenerative potential reflects alterations in the intrinsic capacity for axon growth. Accordingly, understanding the intrinsic mechanisms that regulate axon regeneration provides insights into possible neurologic injuries and disease treatments. However, these intrinsic signaling mechanisms operating in the adult nervous system are not yet fully understood.

Axon regeneration of injured neurons involves reorganization of the cytoskeleton (Bradke et al., 2012). After injury, neurons transform the damaged axon ends into new growth cone-like structures, driven by a dynamic reorganization of the actin network (Coles and Bradke, 2015). The Rho-type GTPase family is central to the regulation of cytoskeletal dynamics, including actin polymerization, F-actin stabilization, and actomyosin assembly in neuronal and non-neuronal cells (Hall, 1998). Rho GTPase functions as a bimolecular switch, with inactive GDP-bound and active GTP-bound forms. Activated Rho GTPases interact with effector proteins that trigger various cellular responses (Bishop and Hall, 2000). Among the Rho GTPase effectors, Rho-associated coiled-coil kinase (ROCK) plays an essential role in actin organization via myosin activation (Amano et al., 1996). Several Rho GTPases, including Rho, Cdc42, and Rac, have been demonstrated to function in the noncanonical Wnt pathway (Schlessinger et al., 2009). This pathway uses universal Wnt signaling components, such as Frizzled receptor (Fz) and Disheveled (Dsh). Although several Wnts have been shown to function in the nervous system, including neuron migration, polarity, neurite extension, and neurite pruning (Whangbo and Kenyon, 1999; Lu et al., 2004; Montcouquiol et al., 2006; Hayashi et al., 2009), the Wnt pathway contribution to axon regeneration is poorly understood.

Recent studies on axon regeneration in genetic model organisms, such as *Caenorhabditis elegans*, have identified common biological pathways that use conserved molecules to control regeneration (Byrne and Hammarlund, 2017). Axon regeneration in *C. elegans* is regulated by the p38 MAP kinase (MAPK), JNK MAPK, and Rho GTPase signaling pathways (Hammarlund and Jin, 2014; Hisamoto and Matsumoto, 2017; Shimizu et al., 2018). The JNK pathway consists of MLK-1 MAPKKK, MEK-1 MAPKK, and KGB-1 JNK; it is inactivated at the KGB-1 activation step by VHP-1, which is a member of the MAPK phosphatase family (Mizuno et al., 2004). The *vhp-1* loss-of-function mutation causes hyperactivation of the JNK pathway, resulting in developmental arrest at an early larval stage. We have previously undertaken a genome-wide RNAi screen for suppressors of *vhp-1* lethality and isolated 92 *svh* genes (Li et al., 2012; Shimizu et al., 2021). Analysis of these *svh* genes was able to shed new light on the regulation of axon regeneration. Moreover, we demonstrated that in *C. elegans*, the RHO-1/RhoA–LET-502/ROCK signaling pathway promotes axon regeneration via phosphorylation of the regulatory nonmuscle myosin light chain [MLC (MLC-4); Shimizu et al., 2018]. We have also found that the *svh-15* gene was previously identified as *brc-2*, which encodes a homolog of the mammalian BRCA2 tumor suppressor (Martin et al., 2005; Shimizu et al., 2018). We have demonstrated that SVH-15/BRC-2 is required for the activation of LET-502 by GTP-bound RHO-1. MLC phosphorylation activates the Mg ATPase activity of nonmuscle myosin II and stimulates the interaction between actin and myosin (Somlyo and Somlyo, 2003). A growth cone contains a specific actin structure, which generates a mechanical force at the leading edge of the lamellipodium (Bradke et al., 2012). A previous study has revealed that myosin II functions in actin-bundle turnover in the growth cones of cultured neurons (Medeiros et al., 2006). These results suggest that MLC-4 phosphorylation regulates the dynamics of regenerating growth cones via the alteration of actin structures. Indeed, in *C. elegans*, MLC-4 is phosphorylated at the ends of the injured axons (Shimizu et al., 2018). The altered phosphorylation state of MLC is attributed to direct ROCK-mediated phosphorylation and inhibition of the myosin-binding subunit of myosin phosphatase (MYPT), which is a part of the MLC phosphatase complex (Totsukawa et al., 2000). Therefore, MYPT is expected to be involved in regulating *C. elegans* axon regeneration.

This study investigated the *svh-16*/*cdk-14* and *svh-21*/*mig-1* genes, which encode homologs of mammalian cyclin-dependent protein kinase-14 (CDK-14) and Fz, respectively. We found that CDK-14 binds to MIG-5/Dsh and EPHX-1/RhoGEF, and showed that D-type motor axons use EGL-20/Wnt and MIG-1/Fz as regulators of axon regeneration. This signal is transduced by the noncanonical Wnt pathway consisting of the CDK-14–EPHX-1–CDC-42–MLC-4 phosphorylation axis.

## Materials and Methods

### 

#### 

##### C. elegans strains.

The *C. elegans* strains used in this study are listed in Table 1. All strains were maintained on nematode growth media and fed with OP50, as described previously (Brenner, 1974).

**Table 1.** Strains used in this study

Strain	Genotype
KU91	*mig-5(km91) dsh-1(ok1445) juIs76 / mIn1[mIs14 dpy-10(e128)] II*
KU501	*juIs76 II*
KU1600	*cdk-14(tm4238) I; juIs76 II*
KU1601	*cdk-14(tm4238) I; juIs76 II; kmEx1601[Punc-25::cdk-14]*
KU1602	*cdk-14(K89R) I; juIs76 II* (line 1)
KU1603	*cdk-14(K89R) I; juIs76 II* (line 2)
KU1604	*mig-5(rh147) juIs76 II*
KU1605	*mig-1(e1787) I; juIs76 II*
KU1606	*juIs76 II; egl-20(n585) IV*
KU1607	*mig-1(e1787) I; juIs76 II; egl-20(n585) IV*
KU1608	*cwn-1(ok546) juIs76 II; cwn-2(ok895) IV*
KU1609	*cdk-14(tm4238) I; juIs76 II; kmEx1406[Punc-25::mlc-4(DD)]*
KU1610	*mig-1(e1787) I; juIs76 II; kmEx1610[Punc-25::mlc-4(DD)]*
KU1611	*cdk-14(tm4238) I; juIs76 II; kmEx1611[Punc-25::rho-1(GTP)]*
KU1612	*cdk-14(tm4238) I; juIs76 II; kmEx1405[Punc-25::let-502*Δ*C]*
KU1613	*cdk-14(tm4238) I; juIs76 II; kmEx1613[Punc-25::cdc-42(GTP)]*
KU1614	*cdk-14(tm4238) I; mel-11(sb56) juIs76/mnC1[dpy-10(e128) unc-52(e444)] II.*
KU1615	*ephx-1(ok494) juIs76 II*
KU1616	*cdk-14(tm4238) I; ephx-1(ok494) juIs76 II*
KU1617	*ephx-1(ok494) juIs76 II; kmEx1405[Punc-25::let-502*Δ*C]*
KU1618	*ephx-1(ok494) juIs76 II; kmEx1613[Punc-25::cdc-42(GTP)]*
KU1619	*cdk-14(tm4238) I; juIs76 II; kmEx1619[Punc-25::ephx-1]*
KU1620	*ephx-1(tm11319) juIs76 II*
KU1621	*cdk-14(tm4238) I; ephx-1(tm11319) juIs76 II*
KU1623	*dsh-2(ok2162) juIs76 / mIn1[mIs14 dpy-10(e128)] II*
KU1624	*mig-1(e1787) I; juIs76 II; kmEx1613[Punc-25::cdc-42(GTP)]*
KU1625	*juIs76 II; kmEx1613[Punc-25::cdc-42(GTP)]*
KU1626	*juIs76 II; kmEx1619[Punc-25::ephx-1]*

##### Plasmids.

The *Punc-25::cdk-14* plasmid was generated by inserting the *cdk-14* cDNA isolated from the pACT cDNA library (Sakamoto et al., 2005) into the pSC325 vector. The *Punc-25::ephx-1* plasmid was generated by inserting the *ephx-1* cDNA (isoform a) isolated from a cDNA library into the pSC325 vector. The *Punc-25::cdc-42(G12V)* plasmid was generated by inserting the *cdc-42(G12V)* cDNA (gift from Kozo Kaibuchi, Nagoya University, JAPAN) and the *cdc-42* 3′UTR into pSC325. The pDBD-CDK-14 plasmid was generated by inserting the *cdk-14* cDNA into pGBDU-C. The pAD-EPHX-1, pAD-EPHX-1N, and pAD-EPHX-1(SH3) plasmids were generated by inserting each cDNA into pACTII. T7-EPHX-1 (full length), and T7-EPHX-1ΔN(1–467) plasmids were generated by inserting the full-length cDNA or the *ephx-1(468–1159)* partial cDNA into pCMV-T7. The 3xFLAG-CDK-14 was generated by inserting the *cdk-14* cDNA into the pCMV-3xFLAG vector. To construct 3xFLAG-CDC-42(T17N), a T17N mutation was introduced into the *cdc-42* cDNA (gift from Kozo Kaibuchi) by PCR, then inserted into the pCMV-3xFLAG vector. The GFP-MIG-5b (MIG-5 isoform b) and GFP-MIG-5b(311–666) plasmids were generated by inserting the full-length and the N-terminal truncated forms of *mig-5b* cDNAs isolated from a cDNA library into the pCMV-GFP vector. The GFP-DSH-1 and GFP-DSH-2 plasmids were generated by inserting the *dsh-1a* and *dsh-2* cDNAs, respectively, isolated from a cDNA library into the pCMV-GFP vector. *Pmyo-2::dsred-monomer* and *Punc-25::venus::mlc-4(DD)* plasmids have been described previously (Li et al., 2012; Shimizu et al., 2018).

##### Generation of the cdk-14(K89R) mutant using CRISPR–Cas9.

The *cdk-14(K89R)* point mutation was generated using the CRISPR–Cas9 system (Sakai et al., 2021a). The CRISPR guide RNA (5′-GAUCUCUUUCAAGGCGACUAGUUUUAGAGCUAUGCU-3′) and single-stranded donor template DNA (5′-ACTAAATTATATTTTCAGACTTGACGGATCTATAGTCGCCTTGCGAGAGATCAAACTTCAATTTCAAGAA −3′) were synthesized [Integrated DNA Technologies (IDT)], and coinjected with the transactivating CRISPR RNA (IDT), *Streptococcus pyogenes* Cas9 3NLS (IDT) protein, and the *Pmyo-2::dsred-monomer* plasmid into the KU501 strain. Each of the F1 animals carrying the transgene was transferred onto a new dish and used for single-worm PCR, followed by DNA sequencing to detect the mutations.

##### Generation of the mig-5(km91) dsh-1(ok1445) mutant using CRISPR–Cas9.

The *mig-5(km91)* mutation was generated using the CRISPR–Cas9 system (Sakai et al., 2021a). The CRISPR guide RNAs (5′-UCUGUUAUUGAAGCCAGAGAGUUUUAGAGCUAUGCU-3′) were synthesized (IDT), and coinjected with the transactivating CRISPR RNA (IDT), *Streptococcus pyogenes* Cas9 3NLS (IDT) protein, and the *Pmyo-2::dsred-monomer* plasmid into the *dsh-1(ok1445) juIs76*/*mIn1[mIs14 dpy-10(e128)] II* strain. Then, each F1 animal carrying the transgene was transferred onto a new dish and used for single-worm PCR, followed by DNA sequencing. The *km91* mutation is a 28 bp insertion that generates a termination codon within the second exon of the *mig-5* gene.

##### Transgenic animals.

Transgenic animals were obtained using the standard *C. elegans* microinjection method (Mello et al., 1991). *Pmyo-2::dsred-monomer*, *Punc-25::cdk-14*, *Punc-25:: venus::mlc-4(DD)*, *Punc-25::*ρ−*1(G14V)*, *Punc-25::cdc-42(G12V)*, and *Punc-25::ephx-1* plasmids were used in *kmEx1601* [*Punc-25::cdk-14* (25 ng/µl) + *Pmyo-2::dsred-monomer* (5 ng/µl)], *kmEx1610* [*Punc-25::venus::mlc-4(DD)* (25 ng/µl) + *Pmyo-2::dsred-monomer* (5 ng/µl)], *kmEx1611* [*Punc-25::rho-1(G14V)* (25 ng/µl) + *Pmyo-2::dsred-monomer* (5 ng/µl)], *kmEx1613* [*Punc-25::cdc-42(G12V)* (25 ng/µl) + *Pmyo-2::dsred-monomer* (5 ng/µl)], and *kmEx1619* [*Punc-25::ephx-1* (25 ng/µl) + *Pmyo-2::dsred-monomer* (5 ng/µl)], respectively. The *kmEx1405* and *kmEx1406* extrachromosomal arrays have been described previously (Shimizu et al., 2018).

##### Microscopy.

Fluorescence imaging of transgenic animals was performed under a 100× objective lens on a fluorescent microscope (model ECLIPSE E800, Nikon) and photographed using a Zyla CCD camera (Oxford Instruments).

##### Axotomy.

Axotomies were performed as described previously (Li et al., 2012). Animals were subjected to axotomy at the young adult stage. Commissures that displayed growth cones or small branches on the proximal fragment were counted as “regenerated.” Proximal fragments that showed no change after 24 h were counted as “no regeneration.” At least 20 individuals with one to three axotomized commissures were observed for most experiments.

##### Measurements of regenerating axons.

The length of regenerating axons for D-type motor neurons was measured using the segmented line tool of ImageJ. Measurements were made from the site of injury to the tip of the longest branch of the regenerating axon. Axons that did not regenerate were excluded. Data were plotted using R (version 4.0.1) and R studio (version 1.3.959).

##### Yeast two-hybrid assays.

The yeast two-hybrid analysis was performed as described previously (Sakamoto et al., 2005). In brief, plasmids were introduced into the *Saccharomyces cerevisiae* reporter strain PJ69-4A (*MATa trp1-901 ura3-52 leu2-3112 his3-200 gal4*Δ *gal80*Δ *Met2::GAL7-lacZ LYS2::GAL1-HIS3 Ade2::GAL2-ADE2*) and cultured on SC-Ura-Leu plates. Transformants were streaked onto fresh SC-Ura-Leu-His plates with 5 mm 5-aminotriazole and incubated at 30°C for 3 d.

##### Biochemical experiments using mammalian cells.

For immunoprecipitation, transfected COS-7 cells were lysed in RIPA buffer [50 mm Tris-HCl, pH 7.4, 0.15 m NaCl, 0.25% deoxycholic acid, 1% NP-40, 1 mm EDTA, 1 mm dithiothreitol, 1 mm phenylmethylsulfonyl fluoride, phosphatase inhibitor cocktail 2 and 3 (Sigma-Aldrich), and protease inhibitor cocktail (Sigma-Aldrich)], followed by centrifugation at 15,000 × *g* for 12 min. The supernatant was added to 10 µl (bed volume) of Dynabeads protein G (Thermo Fisher Scientific) with anti-T7 (catalog #PM022, MBL International Corporation) and anti-GFP (catalog #598, MBL International Corporation) antibodies and rotated for 2 h at 4°C. The beads were then washed three times with ice-cold PBS and immunoblotted using anti-T7 (catalog #T7-Tag, Merck), anti-FLAG (catalog #PM020, MBL International Corporation), and anti-GFP (catalog #JL-8, Clontech) antibodies.

##### Experimental design and statistical analyses.

All experiments were not randomized, and the investigators were not blinded to the group allocation during experiments and outcome assessment. No statistical methods were used to predetermine sample size. Data visualization was performed using Microsoft Excel 2016, R (version 4.0.1), and R studio (version 1.3.959). Statistical analysis was conducted as described previously (Li et al., 2012). Briefly, 95% confidence intervals were calculated using the modified Wald method, and the two-tailed *p* values were calculated using Fisher's exact test on GraphPad QuickCalcs (http://www.graphpad.com/quickcalcs/contingency1/). The Wilcoxon rank-sum test (two tailed) was performed using R (version 4.0.1), R studio (version 1.3.959), and the R exactRankTests package.

##### Homology search, phylogenetic analysis, identification of domains, and alignments of amino acids.

Homology search, identification of conserved domains, and alignments of amino acids were executed using BLAST [Basic Local Alignment Search Tool; National Center for Biotechnology Information (NCBI)], CD-search (NCBI), and Genetyx-Mac programs.

## Results

### SVH-16/CDK-14 is involved in axon regeneration

**Figure 1.**
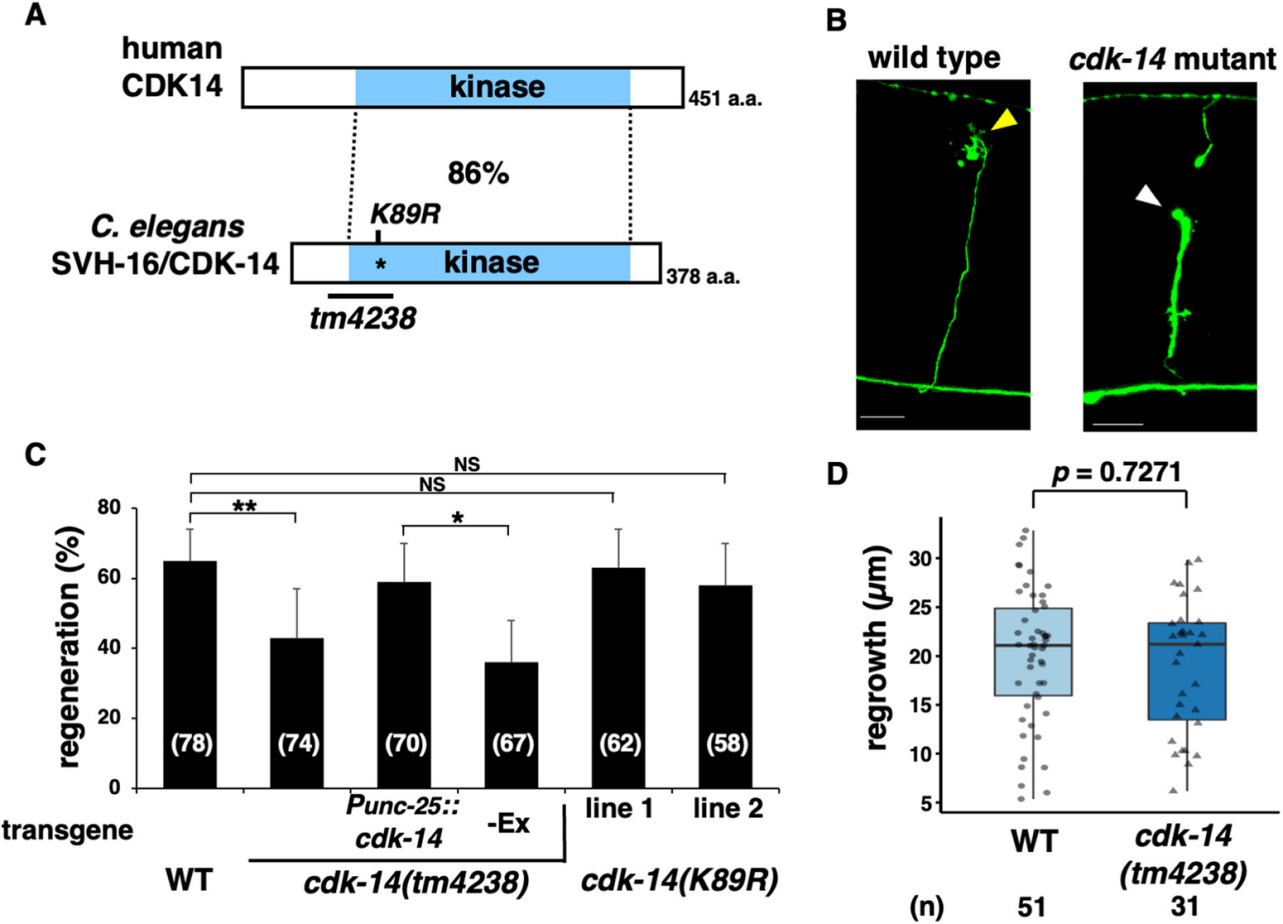
CDK-14 is required for axon regeneration. ***A***, Structure of CDK-14. Schematic diagrams of *C*. *elegans* CDK-14 and human CDK14 are shown. The blue boxes represent the kinase domain. The percentage of amino acid similarity in the kinase domain is shown. The conserved lysine residue (K89) is required for kinase activity. The region deleted in the *tm4238* allele is indicated by the black bar. ***B***, Representative D-type motor neurons in wild-type and *cdk-14* mutant animals 24 h after laser surgery. In wild-type animals, severed axons exhibited regenerating growth cones (yellow arrowhead). In *cdk-14* mutants, the proximal ends of axons failed to regenerate (white arrowhead). Scale bars, 10 µm. ***C***, Percentage of axons that initiated regeneration 24 h after laser surgery in young adults. The number of axons examined is shown. Error bars indicate 95% confidence intervals. *p < 0.05, **p < 0.01, as determined by Fisher's exact test. NS, Not significant. ***D***, Length of regenerating axons 24 h after laser surgery. Data are presented as a boxplot representing median (thick line within the box) and interquartile range (edge of box) with individual data points. The number (*n*) of axons examined is shown. Statistical significance was determined by the Wilcoxon rank-sum test.

So far, we have isolated 92 *svh* genes involved in axon regeneration (Li et al., 2012; Shimizu et al., 2018, 2021). The *svh-16* gene corresponds to Wormbase ORF ZC123.4, which encodes a protein with high homology to mammalian CDK14, a member of the CDK family (Fig. 1*A*; Malumbres, 2014). We, therefore, renamed *svh-16* as *cdk-14*. To clarify the role of *cdk-14* in axon regeneration, we performed laser axotomy and assessed the regeneration of GABA-releasing D-type motor neurons (Fig. 1*B*; Yanik et al., 2004). In young adult wild-type animals, ∼65% of severed axons started to regenerate within 24 h after axonal injury, whereas the frequency was reduced in *cdk-14(tm4238)* mutants (Fig. 1*B*,*C*, Table 2). We investigated the effects of *cdk-14* on growth cone behavior and found that, in *cdk-14(tm4238)* mutants, the length of regenerated axons or axon guidance remains unaffected (Fig. 1*D*). Thus, CDK-14 is explicitly required for initiating axon regeneration. Notably, the effect of *cdk-14* is specific to injury-induced regeneration, as the *cdk-14(tm4238)* mutation did not affect nerve development per se

**Table 2.** Raw data for genotypes tested by axotomy

Strain	Genotype (*juIs76* background)	Axons, *n*	Regenerations, *n* (% of total)	*p* Value	Compared with
KU501*^a^*	wild type	78	51 (65%)		
KU1600	*cdk-14(tm4238)*	74	31 (42%)	0.0055	KU501*^a^*
KU1601	*cdk-14(tm4238); Ex[Punc-25::cdk-14]*	70	41 (59%)		
	*cdk-14(tm4238); Ex[Punc-25::cdk-14] -Ex*	67	24 (36%)	0.0102	KU1601
KU1602	*cdk-14(K89R)* (line 1)	62	39 (63%)	0.8593	KU501*^a^*
KU1603	*cdk-14(K89R)* (line 2)	58	34 (58%)	0.4756	KU501*^a^*
KU1604	*mig-5(rh147)*	68	36 (53%)	0.1328	KU501*^a^*
KU91	*mig-5(km91) dsh-1(ok1445)*	67	43 (64%)	1.0000	KU501*^a^*
KU1623	*dsh-2(ok2162)*	62	40 (65%)	1.0000	KU501*^a^*
KU1605	*mig-1(e1787)*	63	25 (40%)	0.0037	KU501*^a^*
KU1606	*egl-20(n585)*	74	34 (46%)	0.0219	KU501*^a^*
KU1607	*mig-1(e1787); egl-20(n585)*	62	23 (37%)	0.8546 0.3831	KU1605 KU1606
KU1608	*cwn-1(ok546); cwn-2(ok895)*	32	22 (68%)	0.826	KU501*^a^*
KU1609	*cdk-14(tm4238); Ex[Punc-25::mlc-4(DD)]*	50	30 (60%)		
	*cdk-14(tm4238); Ex[Punc-25::mlc-4(DD)] -Ex*	49	18 (37%)	0.0272	KU1609
KU1610	*mig-1(e1787); Ex[Punc-25::mlc-4(DD)]*	56	33 (59%)		
	*mig-1(e1787); Ex[Punc-25::mlc-4(DD)] -Ex*	61	22 (34%)	0.0164	KU1610
KU1611	*cdk-14(tm4238); Ex[Punc-25::rho-1(GTP)]*	61	17 (28%)		
	*cdk-14(tm4238); Ex[Punc-25::rho-1(GTP)] -Ex*	27	11 (41%)	0.3209	KU1611
KU1612	*cdk-14(tm4238); Ex[Punc-25::let-502*Δ*C]*	60	32 (53%)		
	*cdk-14(tm4238); Ex[Punc-25::let-502*Δ*C] -Ex*	66	29 (44%)	0.3724	KU1612
KU1613	*cdk-14(tm4238); Ex[Punc-25::cdc-42(GTP)]*	69	48 (70%)		
	*cdk-14(tm4238); Ex[Punc-25::cdc-42(GTP)] -Ex*	88	32 (36%)	<0.0001	KU1613
KU1624	*mig-1(e1787); Ex[Punc-25::cdc-42(GTP)]*	63	38 (60%)		
	*mig-1(e1787); Ex[Punc-25::cdc-42(GTP)] -Ex*	59	22 (37%)	0.0121	KU1624
KU1625	*Ex[Punc-25::cdc-42(GTP)]*	52	33 (63%)		
	*Ex[Punc-25::cdc-42(GTP)] -Ex*	54	35 (65%)	1.0000	KU1625
KU501*^b^*	wild type	69	42 (61%)		
KU1600*^a^*	*cdk-14(tm4238)*	70	28 (40%)	0.0176	KU501*^b^*
KU1614	*cdk-14(tm4238); mel-11(sb56)*	56	35 (63%)	0.0194	KU1600*^a^*
KU1615	*ephx-1(ok494)*	59	20 (34%)	0.0027	KU501*^b^*
KU1616	*cdk-14(tm4238); ephx-1(ok494)*	73	32 (44%)	0.2846	KU1615
KU1617	*ephx-1(ok494); Ex[Punc-25:::let-502*Δ*C]*	63	32(51%)		
	*ephx-1(ok494); Ex[Punc-25:::let-502*Δ*C] -Ex*	63	25(40%)	0.2828	KU1617
KU1618	*ephx-1(ok494); Ex[Punc-25::cdc-42(GTP)]*	83	51 (61%)		
	*ephx-1(ok494); Ex[Punc-25::cdc-42(GTP)] -Ex*	103	34 (33%)	0.0001	KU1618
KU1619	*cdk-14(tm4238); Ex[Punc-25::ephx-1]*	69	40 (58%)		
	*cdk-14(tm4238); Ex[Punc-25::ephx-1] -Ex*	62	23 (37%)	0.0227	KU1619
KU1626	*Ex[Punc-25::ephx-1]*	54	35 (65%)		
	*Ex[Punc-25::ephx-1] -Ex*	45	28 (62%)	0.8358	KU1626
KU1620	*ephx-1(tm11319)*	64	34 (53%)	0.386	KU501*^b^*
KU1621	*cdk-14(tm4238); ephx-1(tm11319)*	77	47 (61%)	0.0134	KU1600*^a^*

a, b: different controls of the same strain.

**Figure 2.**
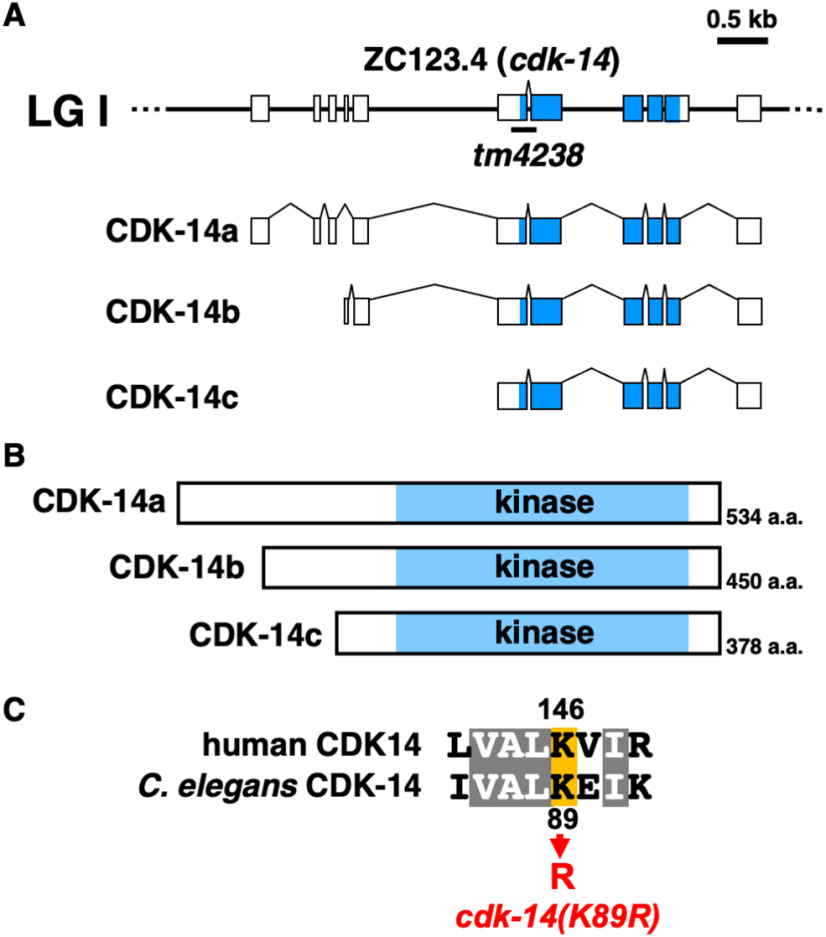
Structure of CDK-14. ***A***, Schematic diagrams of CDK-14 isoforms. The *cdk-14* gene encodes three isoforms: CDK-14a, CDK-14b, and CDK-14c. Boxes indicate exons, and lines indicate introns and untranslated regions. Blue boxes represent the kinase domain. ***B***, Structure of CDK-14 isoforms. Schematic diagrams of CDK-14 isoforms are shown. The blue boxes represent the kinase domain. ***C***, Sequence alignments of human CDK14 and *C. elegans* CDK-14 in the kinase domain. Identical residues are highlighted in gray. The conserved lysine residue (orange) required for kinase activity is shown.

In Wormbase, the genomic sequence of the *cdk-14* gene is predicted to encode three isoforms, CDK-14a, CDK-14b, and CDK-14c (Fig. 2*A*,*B*). To confirm that the *cdk-14* mutation is responsible for the axon regeneration defect, we generated the transgene *Punc-25::cdk-14c*, which contains the *cdk-14c* cDNA and D-type motor neuron-specific *unc-25* promoter. Expression of *cdk-14c* in D-type neurons rescued the defect in axon regeneration observed in *cdk-14(tm4238)* mutants (Fig. 1*C*, Table 2). Therefore, we will hereafter refer to *cdk-14c* as *cdk-14*. Our results indicate that CDK-14 regulates axon regeneration of injured D-type motor neurons after laser axotomy in a cell-autonomous manner.

Mammalian CDK14, also referred to as PFTK1, is activated by a specific interaction with cyclin Y (Jiang et al., 2009). However, the *C. elegans* cyclin Y homolog *cyy-1* is not involved in axon regeneration (Nix et al., 2014). Thus, we hypothesized that the kinase activity of CDK-14 is not required for axon regeneration. To test this possibility, we used CRISPR–Cas9 mutagenesis to generate a catalytically inactive *cdk-14(K89R)* mutant at the endogenous *cdk-14* locus with the invariant Lys-89 mutation in subdomain II, which is critical for ATP binding (Fig. 2*C*; Shu et al., 2007). The *cdk-14(K89R)* mutation had no effect on axon regeneration (Fig. 1*C*, Table 2), suggesting that CDK-14 regulates axon regeneration independent of its kinase activity.

### EGL-20/Wnt–MIG-1/Fz–Dsh signaling pathway regulates axon regeneration

**Figure 3.**
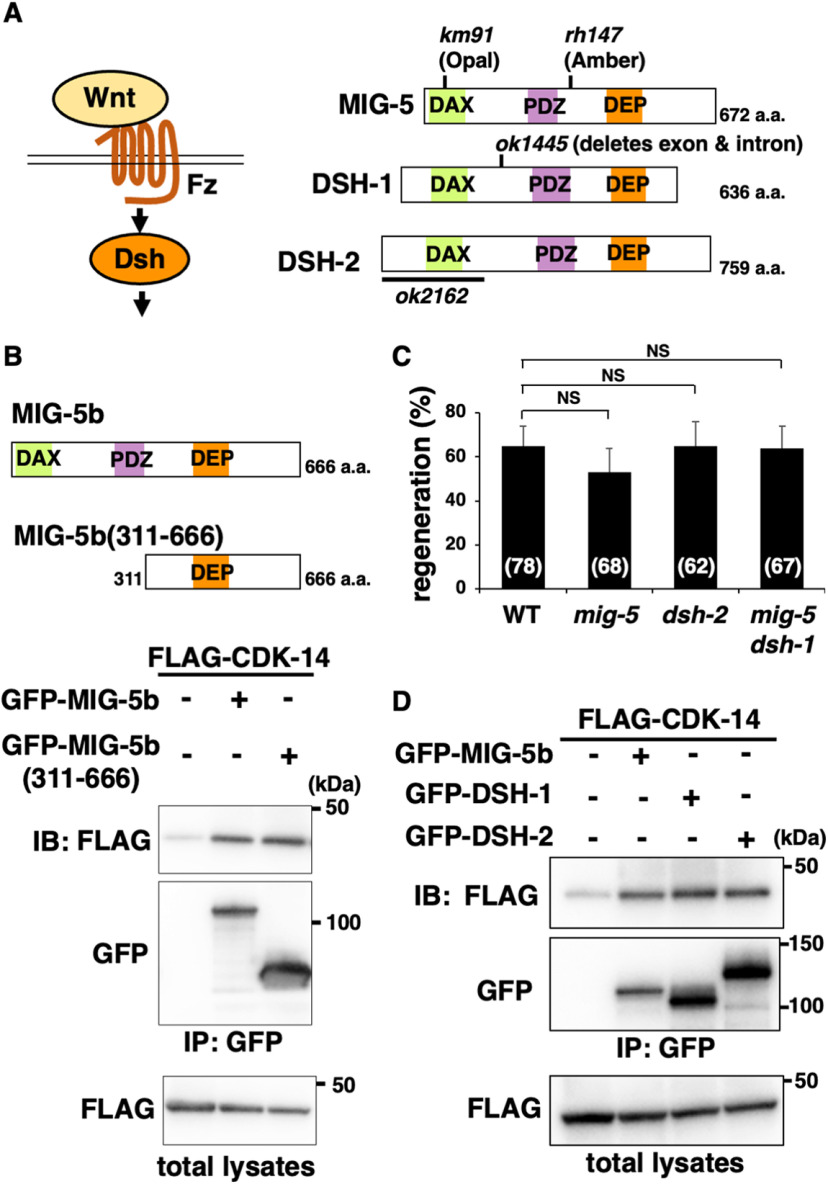
CDK-14 interacts with MIG-5. ***A***, Wnt signaling pathway. Wnt ligand binds to the Fz receptor and activates Dsh. Schematic diagrams of *C*. *elegans* Dsh proteins (MIG-5, DSH-1, and DSH-2) are shown. The *km91*, *rh147*, and *ok1445* mutation sites are indicated. The region deleted in *ok2162* is indicated by black bars. DAX, Domain present in Disheveled and axin; PDZ, PSD-95, Dlg, and ZO-1/2 domains; DEP, Disheveled, Egl-10, and pleckstrin domains. ***B***, Interaction of CDK-14 with MIG-5b. COS-7 cells were transfected with plasmids encoding FLAG-CDK-14, GFP-MIG-5b, and GFP-MIG-5b(311−666), as indicated. Total lysates and immunoprecipitated complexes obtained with anti-GFP antibody [immunoprecipitation (IP): GFP] were analyzed by immunoblotting (IB). Schematic diagrams of MIG-5b and MIG-5b(311−666) are shown above. ***C***, Percentage of axons that initiated regeneration 24 h after laser surgery in young adults. The number of axons examined is shown. Error bars indicate 95% confidence intervals. NS, Not significant. ***D***, Interactions of CDK-14 with DSH-1 and DSH-2. COS-7 cells were transfected with plasmids encoding FLAG-CDK-14, GFP-MIG-5b, GFP-DSH-1, and GFP-DSH-2, as indicated. Total lysates and immunoprecipitated complexes obtained with anti-GFP antibody (IP: GFP) were analyzed by IB.

To understand how CDK-14 regulates axon regeneration, we attempted to identify proteins that interact with CDK-14. We performed a yeast two-hybrid screen using the kinase-negative CDK-14(K89R) as bait and isolated the *mig-5* gene, which encodes a paralog of the Dsh protein (Fig. 3*A*; Walston et al., 2006). This gene product contains a fragment (311−666 aa) of MIG-5 isoform b (Fig. 3*B*). We confirmed the interaction between MIG-5b(311−666) and CDK-14 by immunoprecipitation assays using mammalian COS-7 cells. FLAG-tagged CDK-14 and GFP-tagged MIG-5b(311−666) were coexpressed in COS-7 cells. We immunoprecipitated GFP-MIG-5b(311−666) with anti-GFP antibody and probed for FLAG-CDK-14 by immunoblotting with anti-FLAG antibody. We found that CDK-14 was coimmunoprecipitated with MIG-5b(311−666; Fig. 3*B*) and confirmed that CDK-14 interacts with the full-length MIG-5b (Fig. 3*B*). However, the *mig-5(rh147)* mutation (Fig. 3*A*) did not affect axon regeneration (Fig. 3*C*, Table 2). *C. elegans* has three Dsh paralogs: MIG-5, DSH-1, and DSH-2 (Fig. 3*A*; Walston et al., 2004). Although CDK-14 interacts with DSH-1 and DSH-2 (Fig. 3D), the *dsh-2(ok2162)* and *mig-5(km91) dsh-1(ok1445)* mutations did not cause impaired axon regeneration (Fig. 3*C*, Table 2). Since the *dsh-1(ok1445) dsh-2(or302)* double mutations affected the development of D-type motor neurons, we failed to examine axon regeneration in *dsh-1(ok1445) dsh-2(or302)* mutants. These results suggest that these three Dsh proteins are redundant regulators of axon regeneration.

**Figure 4.**
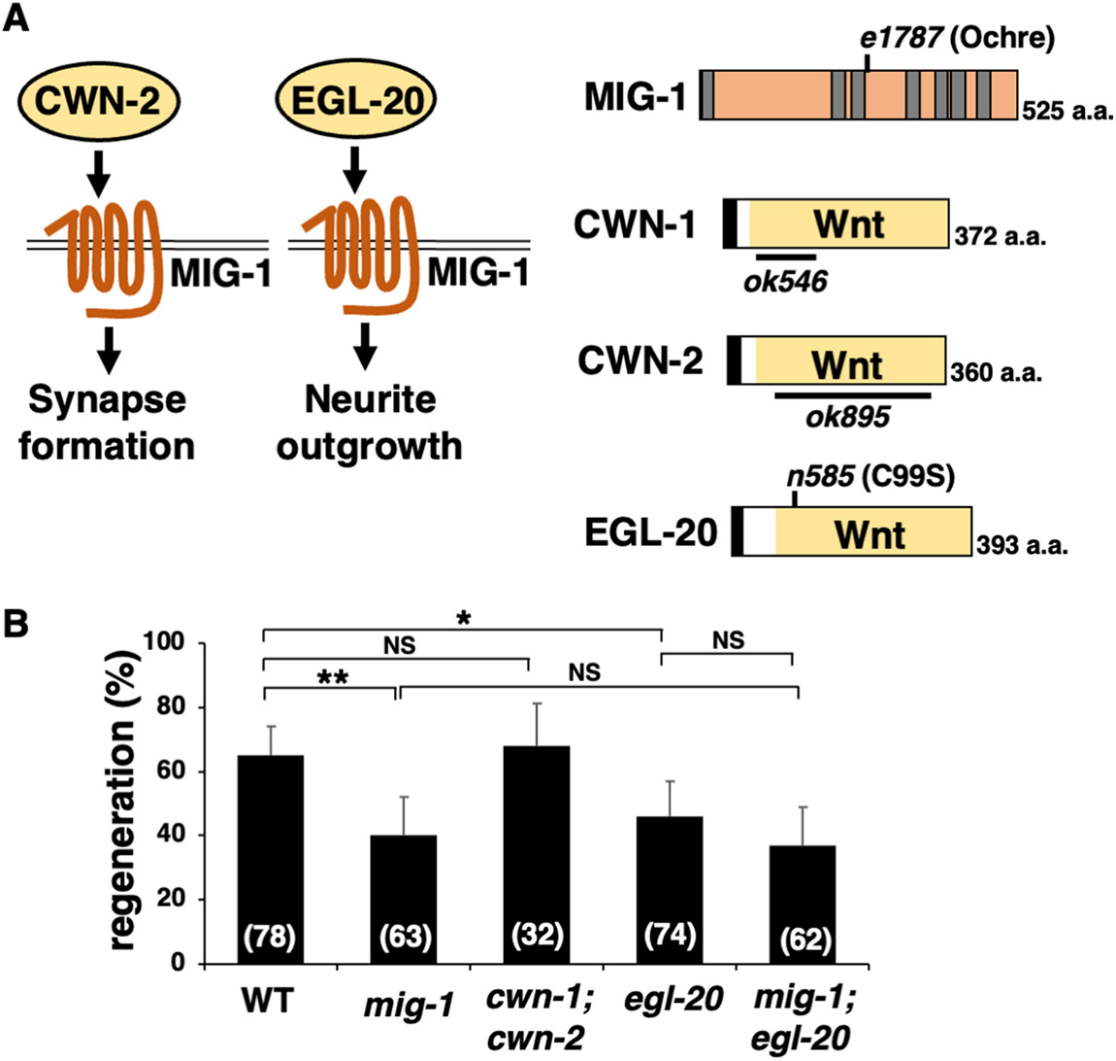
EGL-20/Wnt and MIG-1/Fz are required for axon regeneration. ***A***, MIG-1/Fz-mediated signaling pathways. MIG-1 mediates CWN-2 and EGL-20 signaling to regulate synapse formation and neurite outgrowth, respectively. Schematic diagrams of MIG-1, CWN-1, CWN-2, and EGL-20 are shown. The transmembrane region and signal sequence are indicated by gray and black boxes, respectively. The *e1787* and *n585* mutation sites are indicated. The regions deleted in *ok546* and *ok895* are indicated by black bars. ***B***, Percentages of axons that initiated regeneration 24 h after laser surgery in young adults. The number of axons examined is shown. Error bars indicate 95% confidence intervals. *p < 0.05, **p < 0.01, as determined by Fisher's exact test. NS, Not significant.

As Dsh proteins link Fz receptors to the Wnt signaling pathway (Cadigan and Liu, 2006), we examined the possibility that Wnt regulates axon regeneration. In this pathway, a Wnt ligand binds to the Fz receptor and activates Dsh (Fig. 3*A*). To determine whether the Wnt pathway regulates axon regeneration, we further examined whether there are *svh* genes encoding components that function in the Wnt pathway. We then identified *svh-21*, which is identical to *mig-1*, encoding an Fz family member of *C. elegans* (Fig. 4*A*; Pan et al., 2006). Next, we inquired whether the *mig-1* gene was also involved in axon regeneration, wherein we found that the *mig-1(e1787)* mutation (Fig. 4*A*) significantly reduced axon regeneration after the laser injury (Fig. 4*B*, Table 2). Like *cdk-14* mutants, the *mig-1(e1787)* mutation did not affect axon guidance or nerve development.

*C. elegans* has five Wnts (CWN-1, CWN-2, EGL-20, LIN-44, and MOM-2; Pan et al., 2006), and MIG-1/Fz mediates CWN-2 and EGL-20 signaling to regulate synapse formation and neurite outgrowth, respectively (Fig. 4*A*; Song et al., 2010; Mizumoto and Shen, 2013). We analyzed axon regeneration in *cwn-1(ok546)*; *cwn-2(ok895)* double deletion mutants (Fig. 4*A*). Loss of *cwn-1* and *cwn-2* did not affect axon regeneration (Fig. 4*B*, Table 2). The *egl-20 n585* allele contains a missense mutation that replaces a cysteine at 99 with a serine (Fig. 4*A*); thereafter, a strong reduction-of-function mutation causes severe cell migration defects (Harris et al., 1996; Maloof et al., 1999). We found that the *egl-20* mutant was defective in axon regeneration (Fig. 4*B*, Table 2). Furthermore, the regeneration defect in *mig-1(e1787)*; *egl-20(n585)* double mutants was found to be no more significant than the regeneration defect in single *mig-1(e1787)* or *egl-20(n585)* mutants (Fig. 4*B*, Table 2). Thus, MIG-1 and EGL-20 act in the same pathway. As with the *mig-1* mutant, the *egl-20(n585)* mutation did not affect axon guidance or nerve development. Interestingly, in glutamatergic touch sensory PLM (posterior lateral microtubule) neurons, *egl-20* and *mig-1* have been reported to be involved in axonal regeneration (Chen et al., 2011; Gokce et al., 2017). Thus, these results suggest that the EGL-20/Wnt–MIG-1/Fz pathway is generally required by neurons for axon regeneration.

### CDK-14 acts on the noncanonical Wnt pathway to promote axon regeneration

**Figure 5.**
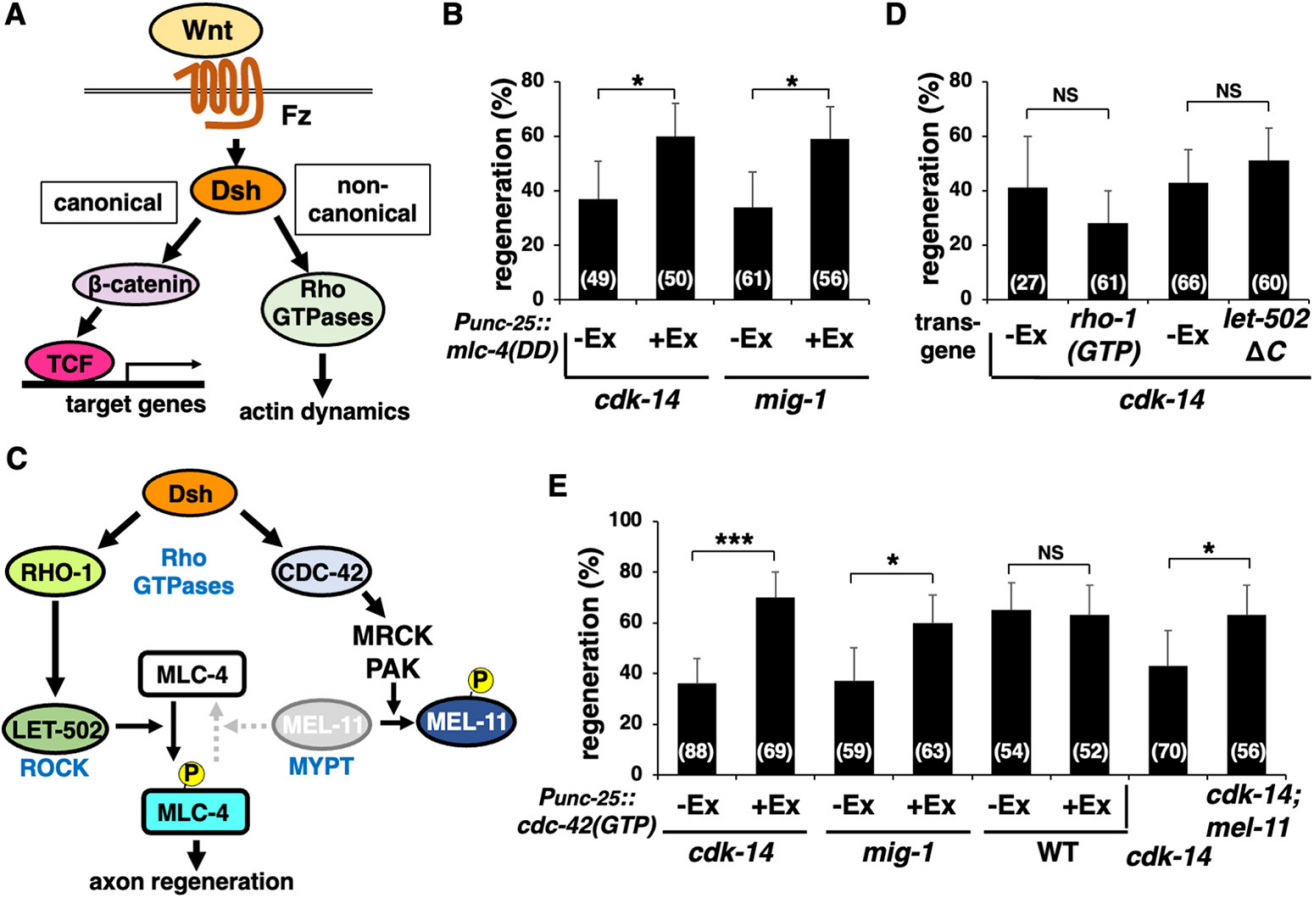
CDK-14 acts on the noncanonical Wnt pathway to promote axon regeneration. ***A***, Canonical and noncanonical Wnt signaling pathways. In the canonical pathway, Wnt stimulation stabilizes β-catenin and induces the expression of TCF target genes. The noncanonical Wnt/pathway activates Rho GTPases and induces reorganization of the actin cytoskeleton. ***B***, ***D***, ***E***, Percentages of axons that initiated regeneration 24 h after laser surgery in young adults. The number of axons examined is shown. Error bars indicate 95% confidence intervals. *p < 0.05, ***p < 0.001, as determined by Fisher's exact test. NS, Not significant. ***C***, Noncanonical Wnt pathways. RHO-1 activates LET-502, which phosphorylates MLC-4. CDC-42 activates MRCK and PAK, which phosphorylate MEL-11 to inhibit myosin phosphatase and induce MLC-4 phosphorylation.

Wnt signaling mediates canonical and noncanonical pathways (Fig. 5*A*; Cadigan and Liu, 2006). The latter pathway requires the activation of Rho GTPases, which regulate cytoskeletal dynamics (Schlessinger et al., 2009). We recently demonstrated that the *C. elegans* RhoA homolog RHO-1 promotes axon regeneration via MLC-4 phosphorylation (Shimizu et al., 2018). To address whether CDK-14 is involved in this pathway, we examined the effect of the phosphomimetic form of MLC-4, MLC-4(T17D; S18D) [termed MLC-4(DD)], on *cdk-14* deficiency in axon regeneration. We found that MLC-4(DD) expression from the *unc-25* promoter suppressed the *cdk-14(tm4238)* mutant phenotype (Fig. 5*B*, Table 2). Similarly, MLC-4(DD) expression suppressed the axon regeneration defect observed in *mig-1(e1787)* mutants (Fig. 5*B*, Table 2). Expression of MLC-4(DD) in wild-type animals does not affect the frequency of axon regeneration (Shimizu et al., 2018). These results suggest that the MIG-1–CDK-14-mediated noncanonical Wnt signaling pathway promotes axon regeneration by inducing MLC-4 phosphorylation.

In the noncanonical pathway, Rho and CDC42 activate ROCK, myotonic dystrophy kinase-related CDC42 binding kinase (MRCK), and p21-activated kinase (PAK; Fig. 5*C*; Gally et al., 2009). These kinases induce myosin II activation by directly phosphorylating MLC (Totsukawa et al., 2000). They also inhibit myosin phosphatase activity by phosphorylating the myosin-binding subunit of MYPT, causing phosphorylation of MLC (Wilkinson et al., 2005). Next, we examined which Rho GTPase participates in the CDK-14-mediated axon regeneration pathway. When active GTP-bound RHO-1(G14V) was expressed from the *unc-25* promoter in *cdk-14(tm4238)* mutants, it failed to suppress the defect in axon regeneration (Fig. 5*D*, Table 2). The *C. elegans* ROCK homolog LET-502 is an effector for RHO-1 (Fig. 5*C*; Piekny et al., 2000). The C-terminal segment of ROCK contains the autoinhibitory Rho-binding domain, the function of which is released via interaction with Rho-GTP (Bishop and Hall, 2000). Accordingly, a LET-502ΔC variant that lacks this C-terminal domain is constitutively active (Shimizu et al., 2018). As with RHO-1(G14V), the expression of LET-502ΔC from the *unc-25* promoter in D-type motor neurons could not suppress the *cdk-14(tm4238)* mutant phenotype (Fig. 5*D*, Table 2). In contrast, the expression of active GTP-bound CDC-42(G12V) from the *unc-25* promoter and the *mel-11(sb56)* mutation defective in MYPT were able to suppress the axon regeneration defect observed in *cdk-14(tm4238)* mutants, respectively (Fig. 5*E*, Table 2). Expression of CDC-42(G12V) did not affect axon regeneration in wild-type animals (Fig. 5*E*, Table 2). Furthermore, we found that expression of CDC-42(G12V) could suppress the axon regeneration defect in *mig-1(e1787)* mutants (Fig. 5*E*, Table 2). Thus, CDK-14 and MIG-1 function upstream of CDC-42, leading to myosin phosphatase inactivation, thereby sustaining MLC-4 phosphorylation.

### EPHX-1/RhoGEF functions between CDK-14 and CDC-42 GTPase in the axon regeneration pathway

**Figure 6.**
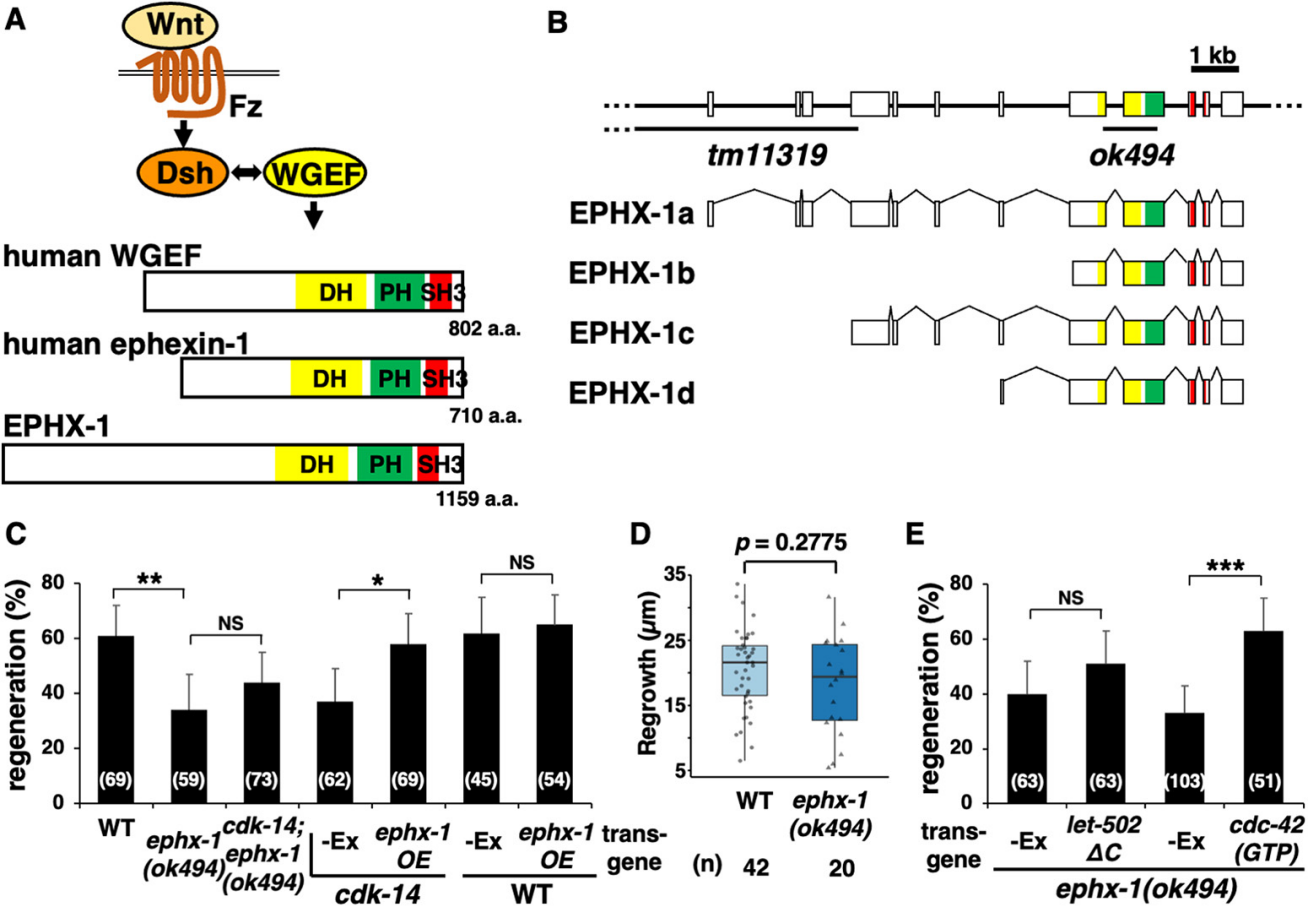
EPHX-1/RhoGEF functions between CDK-14 and CDC-42 GTPase in the axon regeneration pathway. ***A***, Structures of Dbl family proteins. Human WGEF interacts with Dsh in the Wnt signaling pathway. Schematic diagrams of *C*. *elegans* EPHX-1, human WGEF, and human ephexin-1 are shown. ***B***, Structure of the *ephx-1* gene. Schematic diagrams of EPHX-1 isoforms are shown. The *ephx-1* gene encodes four isoforms, EPHX-1a, EPHX-1b, EPHX-1c, and EPHX-1d. Boxes indicate exons, and lines indicate introns and untranslated regions. The yellow, green, and red boxes represent the DH, PH, and SH3 domains, respectively. The regions deleted in *tm11319* and *ok494* alleles are indicated by black bars. ***C***, ***E***, Percentages of axons that initiated regeneration 24 h after laser surgery in young adults. The number of axons examined is shown. Error bars indicate 95% confidence intervals. *p < 0.05, **p < 0.01, ***p < 0.001, as determined by Fisher's exact test. NS, Not significant. ***D***, Length of regenerating axons 24 h after laser surgery. Data are presented as a boxplot representing the median (thick line within the box) and interquartile range (edge of box) with individual data points. The number (*n*) of axons examined is shown. Statistical significance was determined by the Wilcoxon rank-sum test.

Activation of Rho GTPase depends on GEF family members that catalyze the GDP–GTP exchange reaction (Rossman et al., 2005). Therefore, a GEF for the CDC-42 GTPase should function in the noncanonical Wnt pathway to regulate axon regeneration. Human weak-similarity GEF (WGEF) directly interacts with Dsh and functions within the noncanonical Wnt pathway (Fig. 6*A*; Tanegashima et al., 2008). WGEF belongs to the Dbl family of RhoGEF proteins that usually possess tandem Dbl homology (DH), pleckstrin homology (PH), and Src homology 3 (SH3) domains (Fig. 6*A*; Tanegashima et al., 2008). The *C. elegans ephx-1* gene encodes a Dbl family protein homologous to WGEF and ephexin (Fig. 6*A*). We, therefore, examined whether EPHX-1 acts upstream of CDC-42 to regulate axonal regeneration. We found that the *ephx-1(ok494)* mutant (Fig. 6*B*) was impaired in axon regeneration (Fig. 6*C*, Table 2). Like the *cdk-14* mutant, the *ephx-1(ok494)* mutant did not affect the length of the regenerated axons (Fig. 6*D*), axon guidance, or nerve development. Furthermore, the *ephx-1* phenotype defective in axon regeneration was suppressed by the expression of GTP-bound CDC-42(G12V), but not by that of LET-502ΔC (Fig. 6*E*, Table 2). These results support the hypothesis that EPHX-1 functions as a GEF for the CDC-42 GTPase in the noncanonical Wnt pathway to regulate axon regeneration.

Next, we investigated the genetic interaction between *ephx-1* and *cdk-14* in the regulation of axon regeneration. Animals that harbor the *ephx-1(ok494)*; *cdk-14(tm4238)* double mutations exhibited a similar regeneration frequency as the *ephx-1(ok494)* single mutant (Fig. 6*C*, Table 2), indicating that EPHX-1 and CDK-14 act in the same pathway. We then tested the epistatic relationship between *ephx-1* and *cdk-14*. The *ephx-1* gene produces four transcripts from different initiation exons that encode EPHX-1a, EPHX-1b, EPHX-1c, and EPHX-1d (Fig. 6*B*). We constructed the *Punc-25::ephx-1a* fusion gene, in which the *ephx-1a* cDNA was fused with the *unc-25* promoter and found that introducing the *Punc-25::ephx-1a* transgene rescued the defect associated with the *cdk-14(tm4238)* mutation (Fig. 6*C*, Table 2). Overexpression of *ephx-1a* did not affect axon regeneration in wild-type animals (Fig. 6*C*, Table 2). Therefore, overexpression of EPHX-1a from the *unc-25* promoter suppresses the *cdk-14(tm4238)* mutant phenotype. We can conclude that CDK-14 is an upstream component in the EPHX-1–CDC-42 pathway regulating axon regeneration.

### CDK-14 interacts with EPHX-1

**Figure 7.**
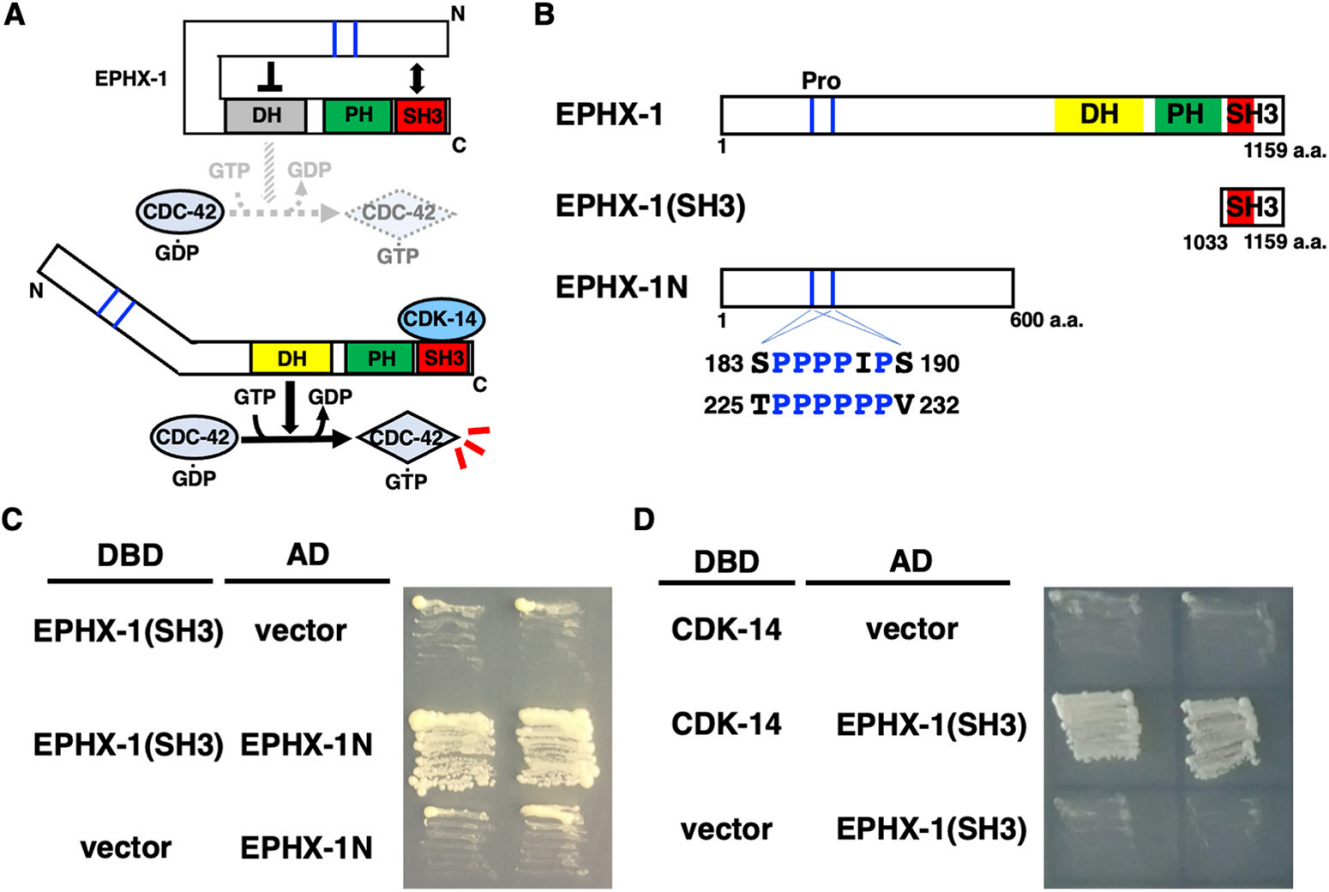
CDK-14 interacts with EPHX-1. ***A***, Model for the regulation of Dbl family member EPHX-1 by intramolecular and intermolecular interactions. In the basal state, the exchange potential of EPHX-1 is autoinhibited by the intramolecular interaction. The SH3 domain binds to the N-terminal region. Intermolecular interaction with CDK-14 disrupts the intramolecular association of the SH3 domain with the N-terminal region. ***B***, Structure of EPHX-1. Schematic diagrams of EPHX-1 are shown. Poly-proline (Pro) regions are indicated. ***C***, ***D***, Yeast two-hybrid assays for the interaction of EPHX-1(SH3) with EPHX-1N (***C***) and CDK-14 (***D***). The reporter strain PJ69-4A was cotransformed with expression vectors encoding GAL4 DBD-EPHX-1(SH3), GAL4 AD-EPHX-1N, and GAL4 DBD-CDK-14 as indicated. Yeasts carrying the indicated plasmids were grown on a selective plate lacking histidine and containing 5 mm 5-aminotriazole for 3 d.

The GEF potential of Dbl family members is autoinhibited by an intramolecular interaction between the N-terminal poly-proline region and the C-terminal SH3 domain (Fig. 7*A*; Schiller et al., 2006). Indeed, the SH3-containing domain of EPHX-1 was associated with the N-terminal domain in the yeast two-hybrid assay (Fig. 7*B*,*C*), and the EPHX-1 N-terminus contains poly-proline regions (Fig. 7*B*). Since CDK-14 regulation of axon regeneration is kinase independent (Fig. 1*C*), we hypothesized that CDK-14 interacts with EPHX-1 to activate EPHX-1 activity by inhibiting intramolecular binding. To investigate this possibility, we asked whether CDK-14 physically interacts with EPHX-1. Yeast two-hybrid analysis revealed that CDK-14 is associated with the SH3-containing (amino acids 1033−1159) domain of EPHX-1 (Fig. 7*D*). These results suggest that the interaction between CDK-14 and the SH3 domain of EPHX-1 disrupts intramolecular binding and activates EPHX-1 in axon regeneration.

### EPHX-1 N-terminal domain inhibits interaction with CDC-42

**Figure 8.**
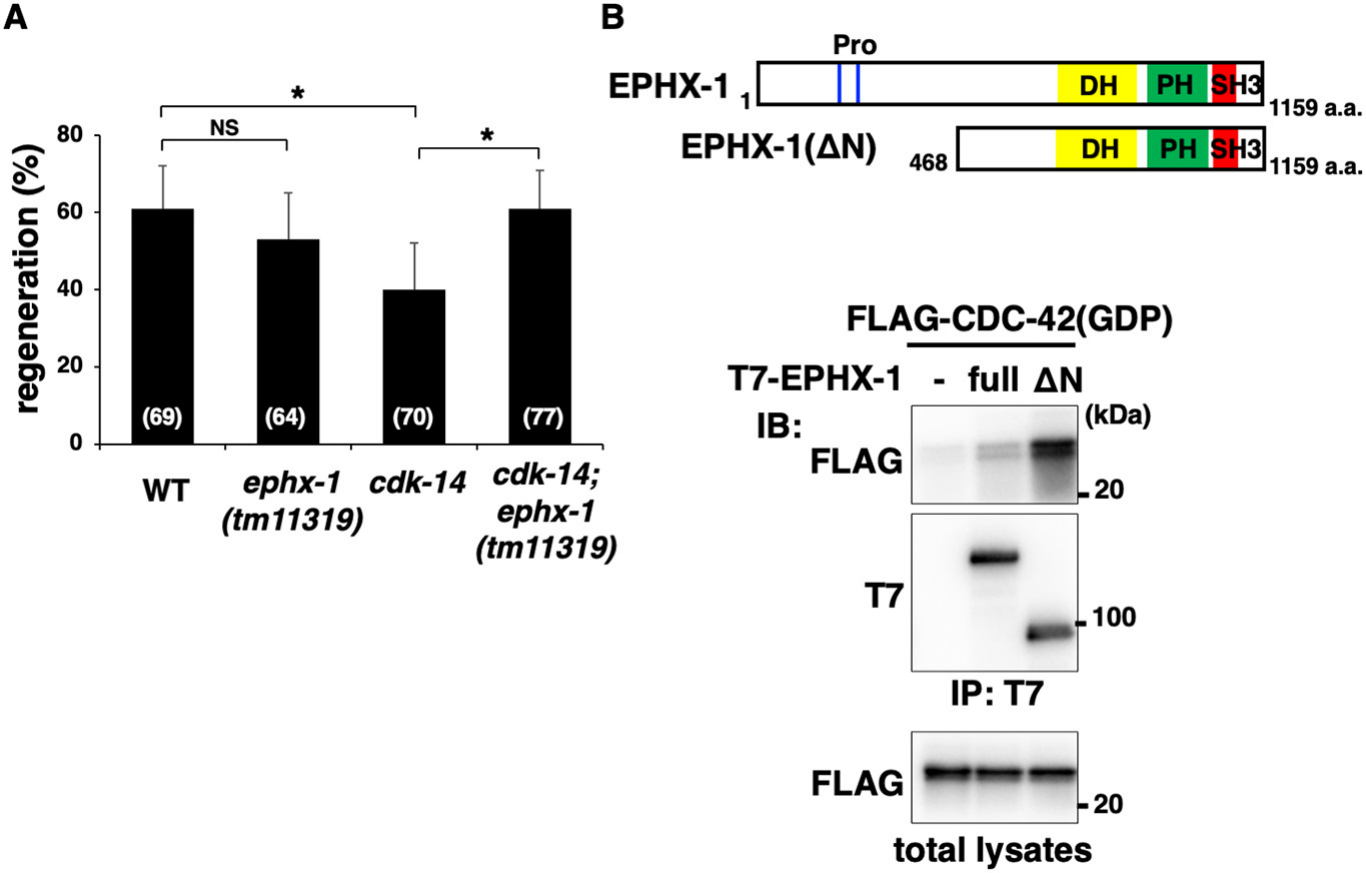
EPHX-1 N-terminal domain inhibits interaction with CDC-42. ***A***, Percentages of axons that initiated regeneration 24 h after laser surgery in young adults. The number of axons examined is shown. Error bars indicate 95% confidence intervals. *p < 0.05, as determined by Fisher's exact test. NS, Not significant. ***B***, Interaction of EPHX-1 with CDC-42. COS-7 cells were cotransfected with T7-EPHX-1 (full length), T7-EPHX-1ΔN(1−467), and FLAG-CDC-42(T17N; GDP) as indicated. Complex formation was detected by immunoprecipitation (IP) with anti-T7 antibody, followed by immunoblotting (IB) with anti-FLAG and anti-T7 antibodies. Total lysates were immunoblotted with anti-FLAG antibody. Schematic diagrams of EPHX-1(full length) and EPHX-1ΔN(1−467) are shown above.

Since the SH3 domain of EPHX-1 interacted with the N-terminal region (Fig. 7*C*), we expected the N-terminal region of EPHX-1 to exert GEF autoinhibitory activity. The *ok494* allele of the *ephx-1* gene encodes a protein with a frameshift deletion between amino acids 719 and 977 of the DH domain, essentially a truncation at amino acid 723 (Fig. 6*B*). The *tm11319* allele has a deletion of the N-terminal domain, resulting in a truncated EPHX-1 (EPHX-1d), with the N-terminal 467 aa deleted (Fig. 6*B***)**. We observed a reduced frequency of axon regeneration in *ephx-1(ok494)* mutants (Fig. 6*C*, Table 2), whereas the *tm11319* mutation had no effect on axon regeneration (Fig. 8*A*, Table 2). These results indicate that the EPHX-1 N-terminal domain is unnecessary for axon regeneration. If the N-terminal region of EPHX-1 inhibits GEF activity, then deletion of the N terminus would be expected to increase EPHX-1 activity. Consistent with this prediction, we found that the *ephx-1(tm11319)* mutation was able to suppress the regeneration defect of *cdk-14(tm4238)* mutants (Fig. 8*A*, Table 2). Therefore, truncation of the N-terminal domain of EPHX-1 can relieve the autoinhibition of GEF activity.

Increased activity of the N-terminal EPHX-1 deletion mutant could be explained by a conformational change in the remaining part of the molecule, affecting its binding to CDC-42. We tested this possibility by analyzing the binding activity of EPHX-1 (full-length) and EPHX-1ΔN(1−467) with CDC-42. EPHX-1ΔN(1−467) corresponds to the EPHX-1d isoform (Figs. 6*B*, 8*B*). We cotransfected FLAG-tagged GDP-bound CDC-42(T17N) and T7-tagged EPHX-1 into mammalian COS-7 cells. Coimmunoprecipitation experiments revealed that EPHX-1ΔN(1−467) had significantly higher CDC-42-binding activity than full-length EPHX-1 (Fig. 8*B*), suggesting that the N-terminal domain of EPHX-1 has an autoinhibitory function.

## Discussion

**Figure 9.**
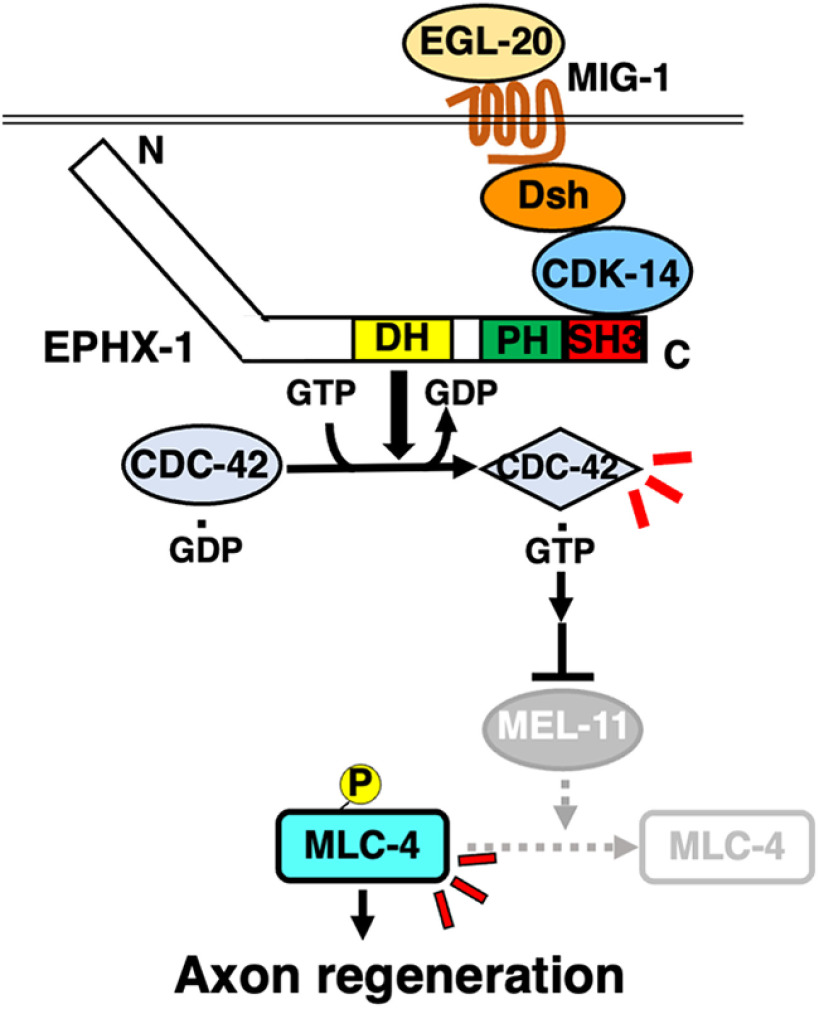
Schematic model for the regulation of axon regeneration by the noncanonical Wnt signaling pathway. The EGL-20/Wnt–MIG-1/Fz signaling promotes axon regeneration by activating the noncanonical Wnt pathway consisting of the CDK-14–EPHX-1–CDC-42–MLC-4 phosphorylation axis. The exchange potential of EPHX-1 is autoinhibited by an intramolecular interaction between the SH3 domain and the N-terminal region. CDK-14 binding to the SH3 domain of EPHX-1 removes the SH3 domain from the N-terminal region, thereby activating the exchange potential of EPHX-1 toward CDC-42. Finally, GTP-bound CDC-42 causes the inactivation of MEL-11, leading to MLC-4 phosphorylation, which promotes axon regeneration.

The Wnt signaling pathway plays multiple roles in the nervous system, including axon guidance, polarity establishment, axon regeneration, and synaptic specificity (He et al., 2018). The importance of Wnt signaling is demonstrated by the conservation of its molecular components across organisms from *C. elegans* to humans. The Wnt pathway is classified into canonical or noncanonical pathways. In the canonical Wnt signaling pathway, Wnt binds to the Fz receptor to activate the transcription of target genes through the regulation of β-catenin and T-cell factor (TCF). The noncanonical Wnt pathway is independent of β-catenin and TCF. This pathway uses universal Wnt signaling components such as Fz and Dsh, but, unlike the canonical Wnt pathway, Dsh functions through Rho family GTPases, subsequently activating downstream JNK to induce cytoskeletal rearrangements (Montcouquiol et al., 2006). Recent studies in mammalian and fish model systems have demonstrated that Wnt signaling promotes axon regeneration after injury in the adult optic nerve and spinal cord (Osakada et al., 2007; Herman et al., 2018), increasing the potential therapeutic value of Wnt. However, our understanding of its downstream signaling pathways remains limited. Our genetic analysis identified a set of Wnt components and demonstrated that EGL-20/Wnt functions in axon regeneration via the Fz receptor MIG-1. Our study reveals that the noncanonical Wnt pathway triggers downstream events that eventually act on the cytoskeleton to initiate axon regeneration (Fig. 9).

How is Wnt signaling transduced from Dsh to Rho GTPase activation in axon regeneration? Our results reveal a mechanistic link between Wnt–Dsh signaling and CDK14. Mammalian CDK14, also called PFTK1, is a member of the CDKs and is activated by a specific interaction with cyclin Y (Jiang et al., 2009). The CDK14/PFTK1–cyclin Y complex activates the noncanonical Wnt pathway, which subsequently activates Rho GTPases, such as Rho, Cdc42, and Rac, resulting in actin polymerization that underlies cell motility and migration (Sun et al., 2014). Analogous to mammalian CDK14/PFTK1, we showed that *C. elegans* CDK-14 functions as an essential regulator of the noncanonical Wnt pathway via activation of CDC-42 in axon regeneration. However, in contrast to mammalian CDK14, the *C. elegans* cyclin Y homolog is not involved in axon regeneration (Nix et al., 2014). Therefore, the kinase activity of CDK-14 is not required for axon regeneration. Consistent with this, a kinase-negative *cdk-14(K89R)* mutation had no effect on axon regeneration, indicating that CDK-14 regulation of axon regeneration is kinase independent.

How does CDK-14 control axon regeneration in a kinase-independent manner? Several studies have shown that the noncanonical Wnt pathway depends on the activation of Rho GTPases (Strutt et al., 1997; Choi and Han, 2002; Habas et al., 2003), which is mediated by RhoGEFs that catalyze the GDP–GTP exchange reaction (Piekny et al., 2000). Indeed, WGEF has been identified in *Xenopus* as a link between Dsh and Rho activation in the noncanonical Wnt signaling pathway (Tanegashima et al., 2008). We also found that *C. elegans* EPHX-1/ephexin RhoGEF mediates noncanonical Wnt signaling in axon regeneration and demonstrated that CDK-14 promotes axon regeneration by activating EPHX-1, which acts on CDC-42. We propose that EPHX-1 is a necessary component in the pathway linking CDK-14 to CDC-42 activation (Fig. 9).

EPHX-1 belongs to the Dbl family of GEFs for Rho GTPases, whose domain structure consists of the DH–PH cassette followed by the SH3 domain (Rossman et al., 2005). The GEF potential of Dbl family members, such as WGEF and ephexin, is autoinhibited by intramolecular and intermolecular interactions (Schiller et al., 2006). The C-terminal SH3 domain binds to the N-terminal region, stabilizing the autoinhibitory helix on the DH domain. This interaction tightly suppresses Rho GTPase access to the DH domain required for GDP–GTP exchange. Indeed, truncation of the N-terminal region of Dbl results in its activation (Eva and Aaronson, 1985). We also showed that the N-terminal region of EPHX-1 inhibits binding to CDC-42. Based on these results, we propose the following mechanism by which CDK-14 activates the GEF activity of EPHX-1. EPHX-1 exchange potential is autoinhibited by an intramolecular interaction between the SH3 domain and the N-terminal region. The binding of CDK-14 to the SH3 domain of EPHX-1 removes the SH3 domain from the N-terminal region, activating the exchange potential of EPHX-1 (Fig. 9).

Biochemical analysis in vertebrates suggests that MLC phosphorylation induces the motility activity of myosin II (Somlyo and Somlyo, 2003). This process involves several Rho GTPase effector kinases, including ROCK, MRCK, and PAK. These kinases phosphorylate MLC directly or inhibit myosin phosphatase activity by phosphorylating MYPT, both of which can induce activation of myosin II (Totsukawa et al., 2000; Somlyo and Somlyo, 2003). These biochemical data suggest two MLC-4 activation pathways. First, LET-502/ROCK directly phosphorylates MLC-4. Second, redundant activities of MRCK and PAK phosphorylate, thereby inactivating MEL-11/MYPT (Piekny et al., 2000). Furthermore, we demonstrated that activated CDC-42 and the defective *mel-11* mutation in MYPT bypass the requirement for CDK-14 in axon regeneration, whereas activated LET-502ΔC cannot, suggesting that CDK-14–EPHX-1–CDC-42 is responsible for myosin II activation via inactivation of MEL-11. Because the *egl-20* gene is expressed in ventral nerve cords (Zhang et al., 2018), it is possible that EGL-20–MIG-1 signaling constitutively activates the CDK-14–CDC-42–MEL-11 inactivation pathway. On the other hand, the RHO-1–LET-502–MLC-4 phosphorylation pathway is activated in response to axon injury (Sakai et al., 2021b). Thus, direct MLC-4 phosphorylation by LET-502 is dependent on axon injury, whereas MLC-4 dephosphorylation is a permissive event.

EPHX-1 functions as a GEF for RHO-1 in axon regeneration regulated by integrin signaling (Sakai et al., 2021b). This indicates that EPHX-1 can act on both RHO-1 and CDC-42 GTPases. How is the specificity of EPHX-1 RhoGEF for cognate GTPases determined? Mammalian ephexin is tyrosine phosphorylated in an N-terminal motif, which then alters the specificity for cognate GTPases. When ephexin is not tyrosine phosphorylated, it activates RhoA, Cdc42, and Rac1; however, it activates RhoA exclusively when the ephexin is phosphorylated (Sahin et al., 2005). Similarly, in *C. elegans*, integrin activation directs the exchange activity of EPHX-1 toward RHO-1 via the SRC-1-mediated tyrosine phosphorylation of the EPHX-1 N-terminal region. These results suggest that tyrosine phosphorylation of EPHX-1 determines the specificity of its GEF activity for RHO-1. Consistent with this, activated LET-502ΔC suppressed the axon regeneration defect caused by the nonphosphorylated *ephx-1(Y568F)* mutation, but not by the *ephx-1* deletion mutation. In the noncanonical Wnt signaling pathway, however, the CDK-14 interaction determines the specificity of EPHX-1 GEF for CDC-42. In this case, the GTP-bound form of CDC-42 suppressed the *ephx-1* deletion defect. One explanation for this result is that the protein kinase activated by active CDC-42 may phosphorylate both MLC-4 and MEL-11. MRCK and PAK are possible kinases that function downstream of CDC-42 (Wilkinson et al., 2005; Gally et al., 2009), but independent mutations in *mrck-1* or *pak-1* did not affect axon regeneration (Chen et al., 2011; Nix et al., 2014). Therefore, MRCK-1 and PAK-1 appear to be redundant kinases that induce MLC-4 phosphorylation during axon regeneration. Although this is the simplest model, we cannot exclude the possibility that a third kinase distinct from MRCK-1 and PAK-1 may work in the axon regeneration pathway. Thus, this calls for further studies to focus on identifying kinases that regulate axon regeneration downstream of CDC-42 in the noncanonical Wnt pathway.
